# Improving the robustness of MOLLI T1 maps with a dedicated motion correction algorithm

**DOI:** 10.1038/s41598-021-97841-z

**Published:** 2021-09-17

**Authors:** Gaspar Delso, Laura Farré, José T. Ortiz-Pérez, Susanna Prat, Adelina Doltra, Rosario J. Perea, Teresa M. Caralt, Daniel Lorenzatti, Julián Vega, Santi Sotes, Martin A. Janich, Marta Sitges

**Affiliations:** 1 MR Applications & Workflow, GE Healthcare, Barcelona, Spain; 2 Universitat de Barcelona, Barcelona, Spain; 3 Hospital Clínic de Barcelona, Barcelona, Spain; 4 MR Applications & Workflow, GE Healthcare, Munich, Germany

**Keywords:** Cardiology, Biomedical engineering

## Abstract

Myocardial tissue T1 constitutes a reliable indicator of several heart diseases related to extracellular changes (e.g. edema, fibrosis) as well as fat, iron and amyloid content. Magnetic resonance (MR) T1-mapping is typically achieved by pixel-wise exponential fitting of a series of inversion or saturation recovery measurements. Good anatomical alignment between these measurements is essential for accurate T1 estimation. Motion correction is recommended to improve alignment. However, in the case of inversion recovery sequences, this correction is compromised by the intrinsic contrast variation between frames. A model-based, non-rigid motion correction method for MOLLI series was implemented and validated on a large database of cardiac clinical cases (n = 186). The method relies on a dedicated similarity metric that accounts for the intensity changes caused by T1 magnetization relaxation. The results were compared to uncorrected series and to the standard motion correction included in the scanner. To automate the quantitative analysis of results, a custom data alignment metric was defined. Qualitative evaluation was performed on a subset of cases to confirm the validity of the new metric. Motion correction caused noticeable (i.e. > 5%) performance degradation in 12% of cases with the standard method, compared to 0.3% with the new dedicated method. The average alignment quality was 85% ± 9% with the default correction and 90% ± 7% with the new method. The results of the qualitative evaluation were found to correlate with the quantitative metric. In conclusion, a dedicated motion correction method for T1 mapping MOLLI series has been evaluated on a large database of clinical cardiac MR cases, confirming its increased robustness with respect to the standard method implemented in the scanner.

## Introduction

Cardiovascular Magnetic Resonance (CMR) imaging is a noninvasive cross-sectional imaging tool for the assessment of detailed information about the anatomical structure and function of the cardiovascular system. It has an important role in the diagnosis of ischemic heart disease, congenital heart disease, cardiomyopathies and myocarditis, as well as in the detection of myocardial edema and determination of viability^1^.

The term “quantitative” in a CMR study is used when the results of a measurement can be expressed in physical units^2,3^ and compared across subjects and sites. Typically, these results are images mapping the value of the physical or chemical variable of interest. Specifically, quantitative relaxation mapping techniques are being used with increasing success due to their ability to deliver repeatable, objective diagnostic criteria based on non-invasive tissue characterization.

Such is the case of myocardial T1 mapping, which allows the detection of heart muscle abnormalities and constitutes a reliable indicator of several cardiomyopathies related to changes of myocardial extracellular content (e.g. edema, fibrosis) as well as fat, iron and amyloid content^4–8^. Currently, it is used not only as a diagnostic imaging tool, but also for treatment monitoring and prognosis.

Spin–lattice relaxation time (T1) is a fundamental property of hydrogen nuclei protons within an established magnetic field and measures how quickly the net magnetization vector recovers to its ground state in the direction of the main magnetic field^9^. Several pathological processes alter the composition of the tissue, leading to changes in the T1 value, which can be used to characterize the degree of myocardial alteration^10^. Some of the pathologies that have been reported to be related to elevated T1 relaxation time are acute and chronic myocardial infarction, myocarditis, amyloidosis, lupus and system capillary leakage syndrome. Regarding lower T1 relaxation time, it has been associated with Anderson Fabry disease and iron overload, among others. When acquired before and after the administration of contrast agent, T1 information can be used to calculate the extracellular volume (ECV) fraction, another clinical marker of myocardial remodeling^11^.

T1-mapping techniques typically rely on pixel-wise fitting an exponential model to a series of CMR measurements. Images are acquired at multiple time points on the recovery curve and the T1 relaxation time is estimated to produce a representation of the magnetic properties of tissue, commonly viewed as a color-coded map of the heart.

To obtain accurate T1 estimates, good anatomical alignment between the measurements is required. Electrocardiogram (ECG) triggering is used to ensure that images are acquired at the same cardiac phase and patients are instructed to hold their breath to ensure consistent respiratory position. However, it is not uncommon to see displacements between frames, caused by cardiac and respiratory triggering inaccuracies, as well as by patient bulk motion. These constitute an important obstacle to obtain accurate T1 estimates and can in extreme cases lead to misdiagnosis^12^. Motion correction methods are recommended to minimize this effect^13^.

**Figure 1:**
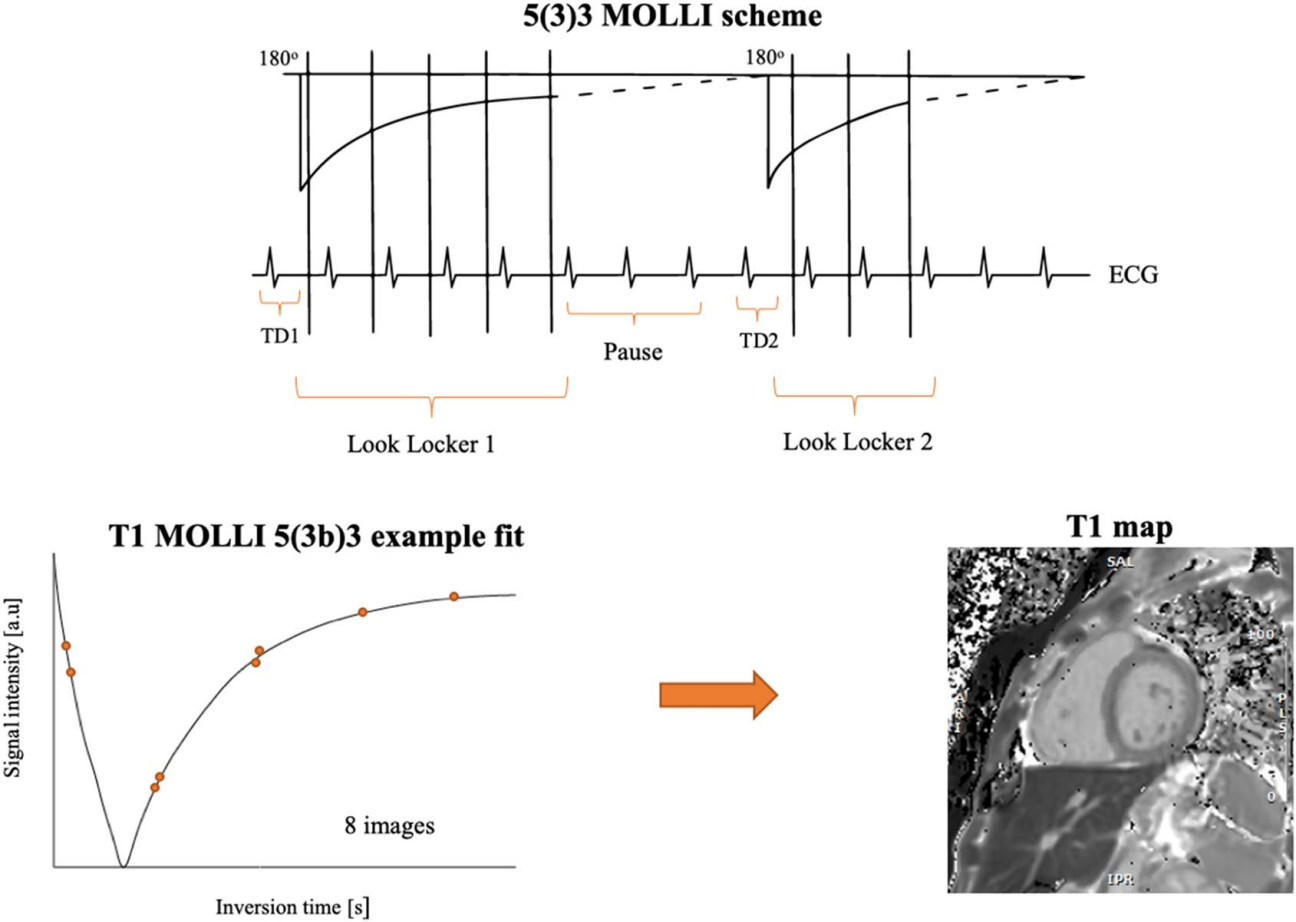
Schematic representation of the MOLLI acquisition pattern and subsequent T1 mapping by pixel-wise exponential fitting.

In the case of T1-mapping based on modified Look-Locker inversion recovery (MOLLI) sequences^14^, image registration is compromised by the different contrast properties of the frames constituting the relaxation series. As it can be observed in Fig. 1, the MOLLI sequence consists of multiple Look-Locker inversion pulses^15^ over several heartbeat cycles, to acquire a series of images with varying inversion times (TI). This scheme allows precise T1 mapping of the myocardium within one breath-hold but results in image series with rapidly changing tissue-dependent contrasts. Specifically, image frames where myocardium and blood pool intensities coincide (i.e. corresponding to inversion times where their respective T1 recovery curves intersect) are particularly prone to misregistration, due to the lack of edge information to constrain the deformation optimization.

In this study, we present the validation of a motion correction method designed to account for the contrast properties of MOLLI data. In particular, the similarity metric compensates for the intensity changes caused by T1 magnetization relaxation between measurements. The proposed method was applied to a large database of clinical CMR cases, with the purpose of determining its robustness when applied to pathological cases, where anatomical alignment is often impaired by arrythmia episodes and breath-hold difficulties.

## Methods

All methods were performed in accordance with the relevant guidelines and regulations. A cohort of 186 patients referred for a CMR at Hospital Clínic (Barcelona, ES) was included in this study (115 male/71 female; weight 75 ± 15 kg; age 55 ± 16 years). All acquisitions were performed on a 3.0 T GE Signa Architect (General Electric Healthcare, Chicago, IL) scanner running software version DV26.0 R02 1810.b. The acquired protocol included one or more MOLLI series, with the following sequence parameters: 2D balanced steady-state free precession (bSSFP), matrix 160 × 148, phase field-of-view 0.8–1.0, pixel size 1.4 × 1.4 mm^2^, slice thickness 8 mm, echo time 1.4 ms, repetition time 3.0 ms, flip angle 35°, 1 average, bandwidth 100 kHz, acceleration 2 × ASSET (Array coil Spatial Sensitivity Encoding). The acquisition pattern was 5(3s)3, i.e. five images acquired in consecutive heartbeats, then a three-second pause for magnetization recovery followed by the acquisition of three more images (see Fig. 1). The acquisitions were performed using the 16-element Anterior Array and 40-element Posterior Array in Body-48 configuration. This resulted in 1133 MOLLI series to be independently analyzed. The raw data of all acquisitions was exported for offline processing.

A prototype reconstruction algorithm, implemented using GE’s Orchestra libraries, was used to retrospectively reconstruct all the exported raw data. The reconstruction consisted of a standard 2D Cartesian pipeline, including an optional post-reconstruction frame-by-frame motion correction step. The results were reviewed by two board-certified cardiologists with 2 and 3 years of experience in CMR reading and T1 mapping.

In its standard implementation, the motion correction step consists of three iterations, in which all frames of the MOLLI series are independently registered to a common reference, typically the median of all frames. An elastic deformation model is used, with a cross-correlation (weight 1, radius 7) similarity metric, implemented using the Advanced Normalization Tools^16^ open-source library. The optimization parameters are: resolution levels 4, gradient step length 0.2, delta time 0.01, number of time steps 100. A Gausssian regularizer with sigma 7 was applied to both the similarity gradient and deformation field.

Additionally, a new correction algorithm was implemented, based on a similarity criterion that accounted for the relaxation properties of the tissue. Rather than using a fixed median frame as reference, a reference series was created by simulating the inversion recovery of the pixels in the first frame at the inversion times where the rest of frames were acquired (see Suppl. Fig. 1). For this purpose, the parameters of an exponential inversion recovery model (*y* = *Ae*^*−t/T1*^ + *C*) were first obtained by non-linear least squares fitting of the previous iteration results. The zero-crossing time was automatically estimated to account for signal phase. Three iterations of frame-wise nonrigid registration were then performed. The first iteration used the more robust standard reference, and the second and third iterations used the new series, thus ensuring that each frame was registered to a reference with similar contrast properties. The parameters of the elastic registration were equivalent to the ones for the standard algorithm.

Due to the large number of datasets analyzed, an automated metric was required to quantify the performance of the different motion correction options. In general, motion correction algorithms are based on the optimization of a data alignment metric, in this case the mutual information between frames. However, it is difficult, based on the numerical value reached by this metric at the end of the optimization, to draw conclusions about the quality of the parameter maps that will be obtained. For this reason, we employed a separate metric to validate the results of the motion correction, not in terms of image alignment, but in terms of the quality of the resulting parameter map.

The reconstructed data were automatically analyzed to determine the quality of the registration results. The coefficient of determination (R^2^) of the voxel-wise T1 fitting of the reconstructed image series was used to assess the performance of both registration methods:

$${R}^{2}=1-\frac{{SS}_{res}}{{SS}_{tot}}$$

where *SS*_*tot*_ represents the total sum of squares of the samples and *SS*_*res*_ the sum of squares of the fitting residuals.

A quantitative metric of anatomical alignment quality, designated Accurate Fitting Prevalence (AFP), was defined as the percentage of voxels with high R^2^ on a manually defined region of interest Ω containing the heart:

$$AFP=100 \left(\frac{1}{\left|\Omega \right|}\sum_{i\in \Omega }\left({R}_{i}^{2}>0.95\right)\right)$$

The AFP metric was used to automatically compare the results obtained with the new motion correction method, with conventional motion correction as implemented in the scanner, and with the uncorrected MOLLI series.

To confirm the validity of the AFP quantitative metric as a surrogate of anatomical alignment, a qualitative evaluation was performed on a subset of cases. The qualitative evaluation consisted in visually scoring a randomly selected set of 200 uncorrected series, using the three-class scoring scale defined in Table 1.

**Table 1:** Qualitative data alignment scoring scale.

Score	Motion	Definition
1	No visible motion	No movement can be perceived
2	Minor motion	Displacements of less than the half of the myocardium thickness in a zone of the heart
3	Major motion	Displacements of more than the half of the myocardium thickness in a zone of the heart or in all the heart

### Ethics approval and consent to participate

This study was approved by the Ethics Evaluation board of the Hospital Clínic of Barcelona. Enrolled subjects provided signed informed consent to participate. All methods were performed in accordance with the relevant guidelines and regulations.

## Results

All data were successfully reconstructed with three methods: uncorrected; with the standard correction method included in the scanner; and with the new dedicated method for T1 mapping. Qualitatively correct T1 maps were generated automatically in all cases.

**Figure 2:**
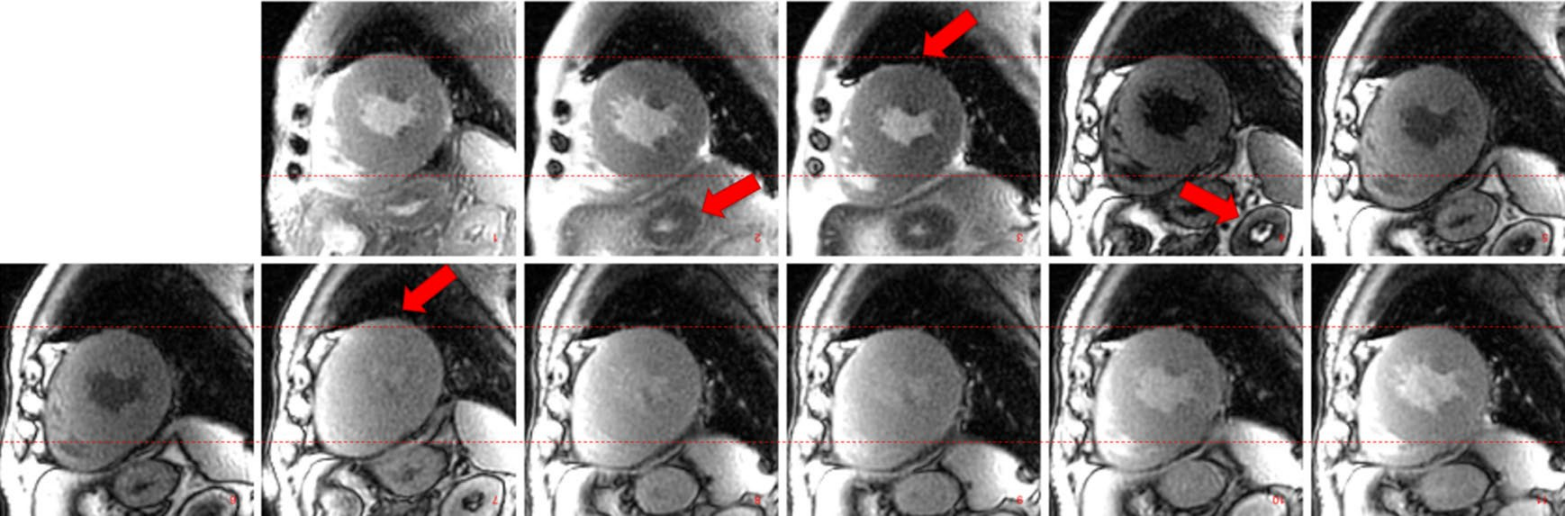
Example cardiac MOLLI series acquired on a 3 T Signa Architect, illustrating the changing tissue contrast. The red arrows indicate anatomical structures affected by motion, compromising T1 mapping accuracy.

Figure 2 shows a typical MOLLI series used for T1 mapping. The red arrows indicate the anatomical structures affected by motion, which would lead to locally incorrect T1 estimates. Reconstruction times (including motion correction) were equivalent with the new method and that implemented in the scanner, under two minutes for a 3-slice 512 × 512 5(3s)3 MOLLI series.

**Figure 3:**
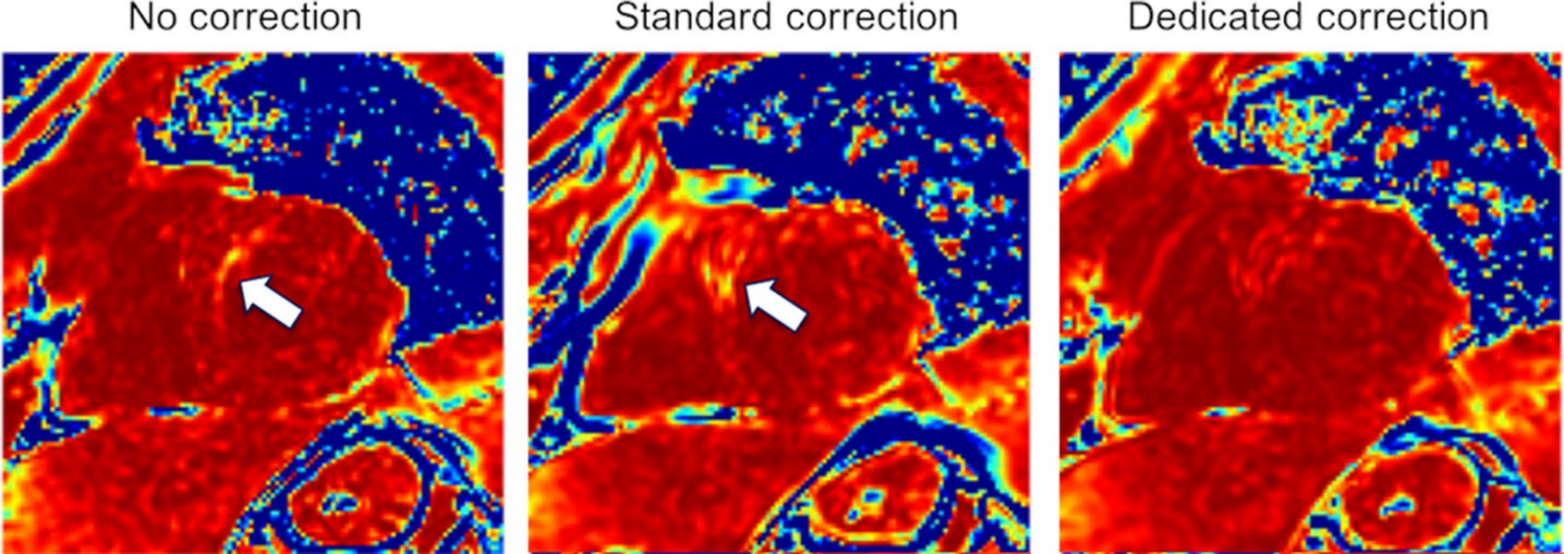
Coefficient of determination (R^2^) maps of the T1 maps obtained from the same MOLLI series, using the three motion correction approaches compared in this study. Values from 0.95 to 1.00 are shown. The arrows indicate regions where myocardial motion resulted in decreased T1 accuracy, aggravated by misregistration with the standard correction method. These maps were created using Matlab (MathWorks, Natick, MA).

Overall, the voxel-wise maps of the coefficient of determination showed improvement with either motion correction, in comparison to the uncorrected reconstruction. However, as can be seen in Fig. 3, errors caused by motion were in some cases exacerbated by image registration failures. This issue, typically found with the standard motion correction, was avoided in most cases by the dedicated correction method. Motion correction caused noticeable (i.e. > 5%, based on the inter-class distances appreciated in Fig. 7) performance degradation in 12% of cases with the standard method, compared to 0.3% with the new one.

**Figure 4:**
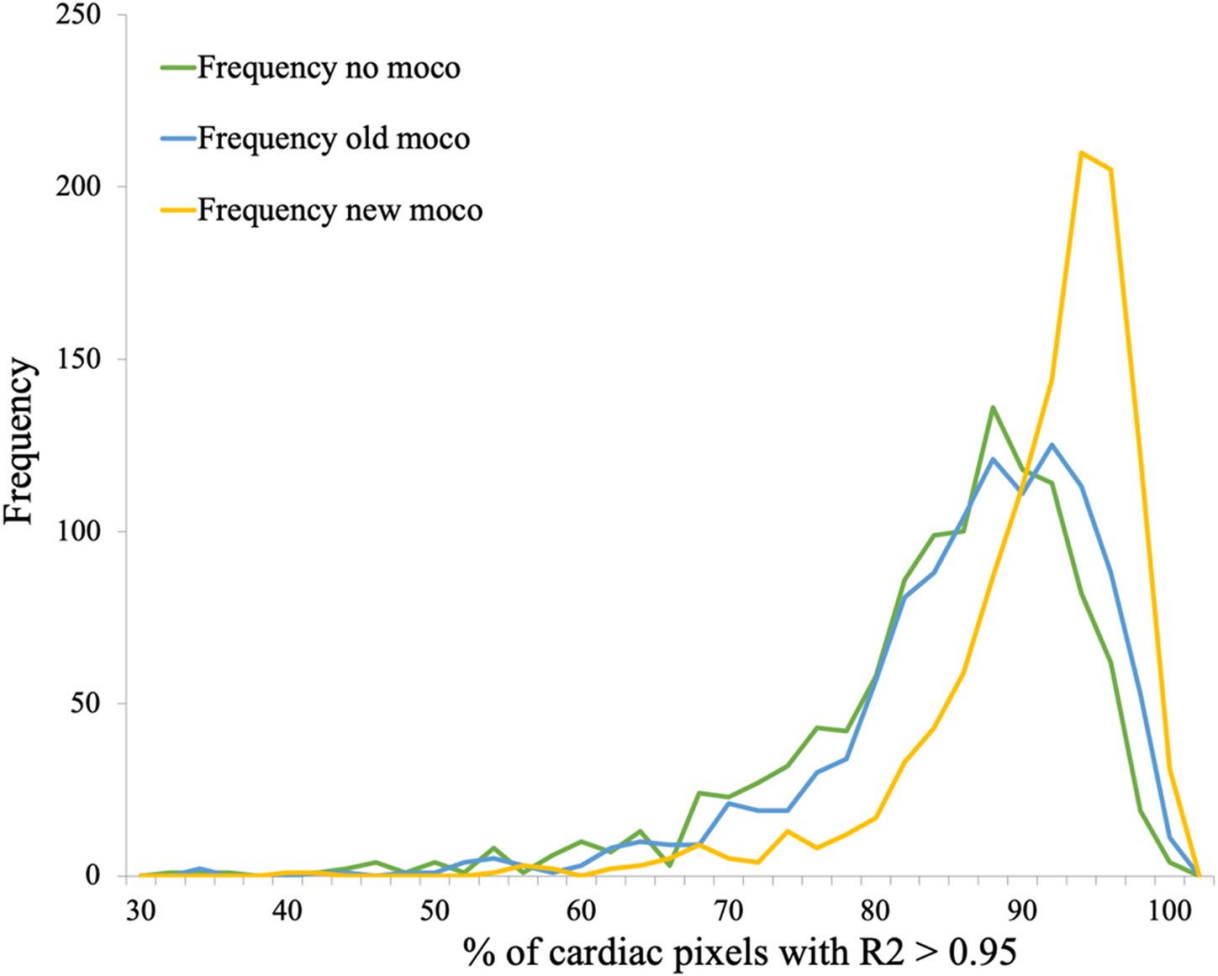
Histograms of the AFP values obtained on the entire patient database with each of the three motion correction approaches. Notice the clear improvement with the new method, resulting in a peak shift of ~ 5%, confirmed by the qualitative analysis to have a visual impact. These graphs were created using Matlab (MathWorks, Natick, MA).

The average AFP of the 1133 cases analyzed was 85% ± 9% with the default motion correction algorithm and it increased to 90% ± 7% with the new dedicated method. In terms of AFP improvement with respect to uncorrected data, the standard method yielded + 3% ± 8% and the new method + 9% ± 8%. Figure 4 shows the AFP histogram, over all datasets, obtained with the three different reconstructions.

**Figure 5:**
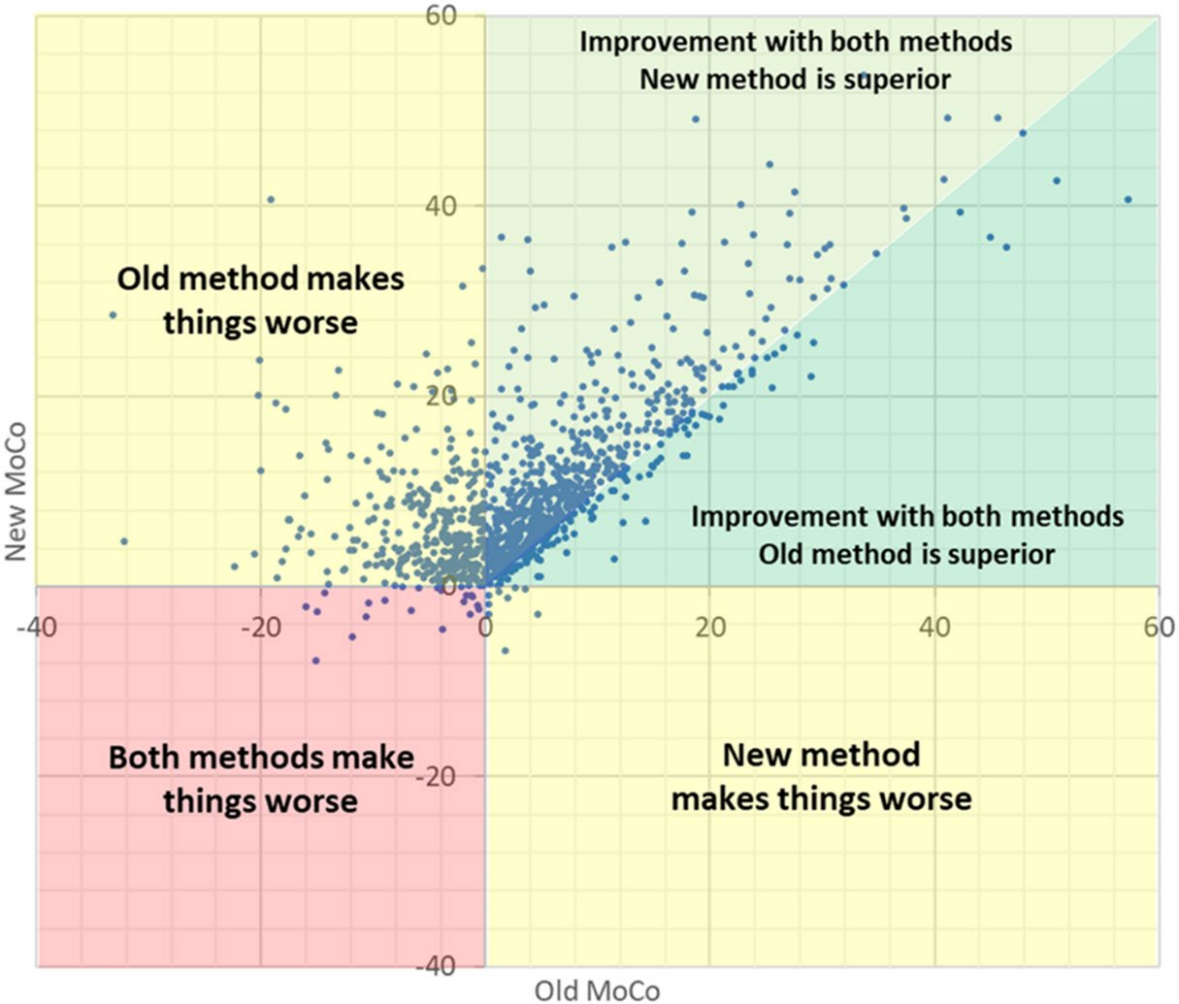
Scatter plot illustrating the relative change in AFP metric obtained with each of the motion correction methods, compared to uncorrected data. Graph created using Excel (Microsoft, Redmond, WA).

The relative performance of the different methods can be appreciated in Fig. 5. The scatter plot shows the distribution of the datasets in four main groups: Quadrants III and IV contain a relatively small number of cases (2%) where the new motion correction method causes performance degradation in comparison with uncorrected data. In contrast, quadrants II and III contain a considerable number of cases (31%) where the standard correction leads to performance degradation. Quadrant I contains the datasets where either motion correction yields a performance improvement, with a vast majority of cases showing the new method outperforming the standard one (59%), with only a few cases (10%) where the new method performs worse than the standard one.

**Figure 6:**
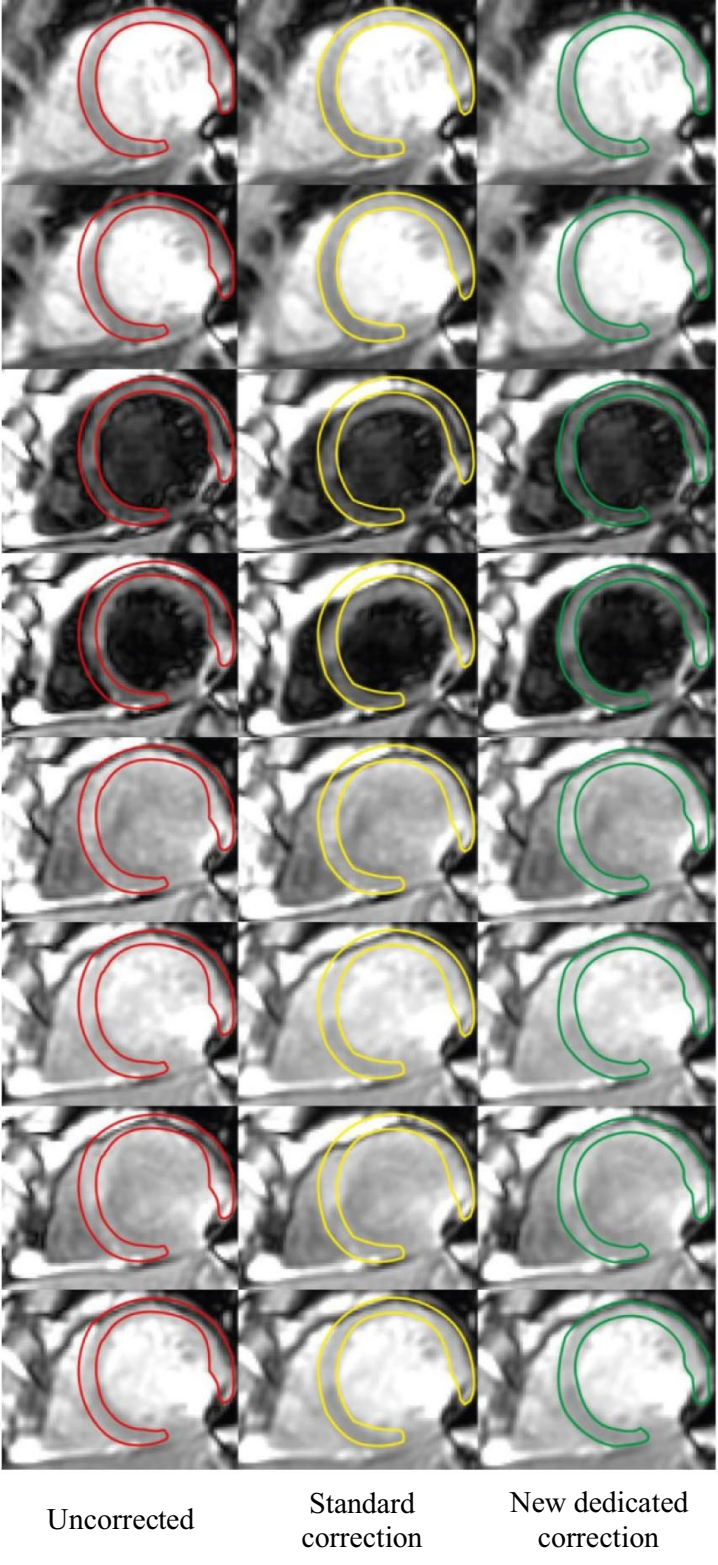
Example MOLLI series, processed with each of the three motion correction approaches discussed in this study. The myocardial delineation of the first frame is overlaid on all frames for reference. Notice how, in this case, the standard correction approach has resulted in misregistration of frames 3 to 5.

A representative example of the improvement of anatomical alignment obtained with the new dedicated method is shown in Fig. 6.

The results of the visual analysis were found to correlate with the quantitative metric. The mean AFP value for each of the three data alignment categories was, respectively: 89.6% ± 7.3% for the “no visible motion” class; 84.9% ± 6.5% for the “minor motion” class; and 74.5% ± 10.6% for the “major motion” class. The differences between class averages were found to be statistically significant with P < 10^–2^. It can be observed that inter-class distance is approximately 5%. In the rest of the analysis, AFP differences below this threshold were interpreted as visually imperceptible.

**Figure 7:**
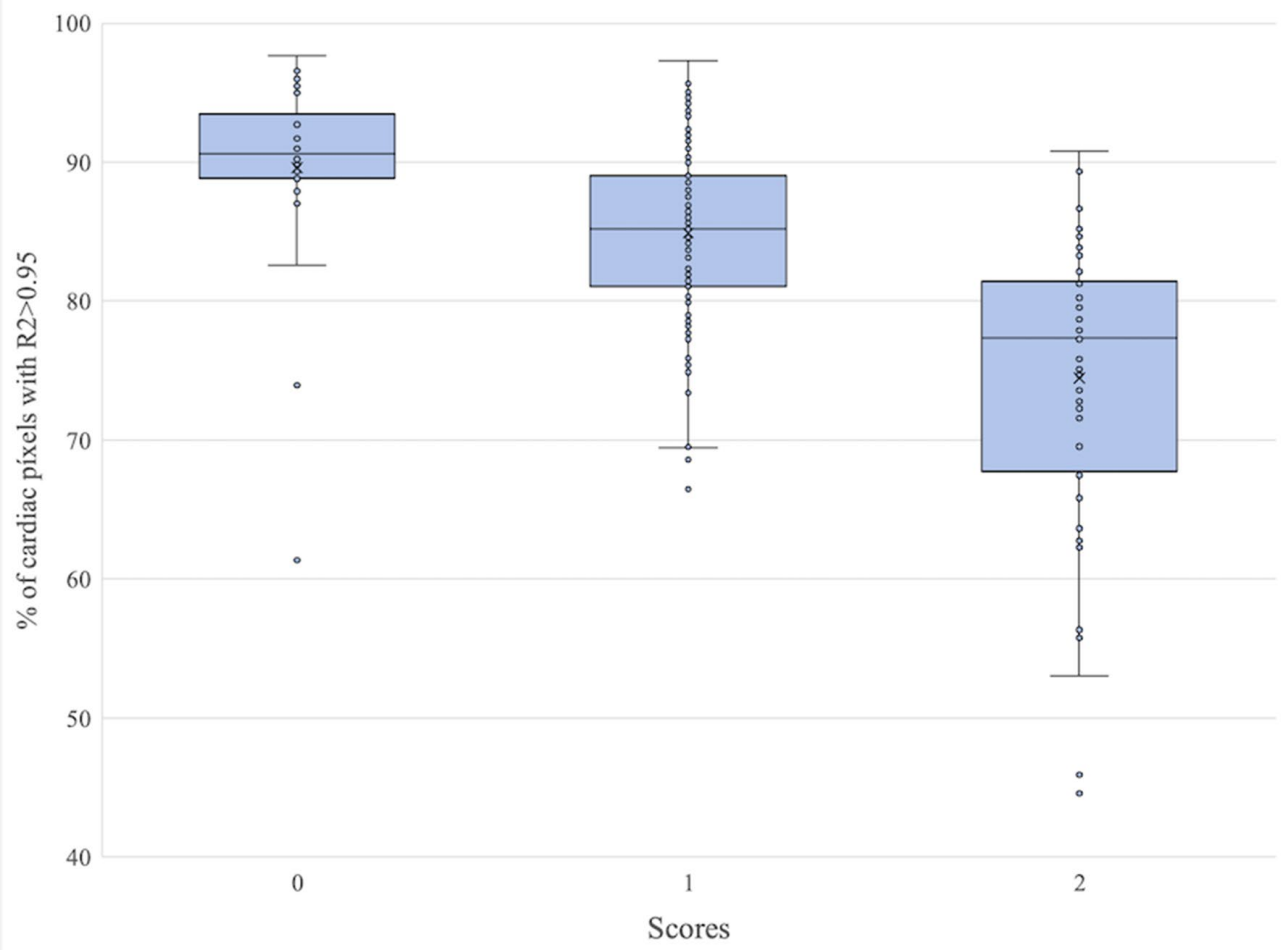
Box and whisker plot of the qualitative evaluation results, showing how the three groups resulting from the visual scoring correlate with the AFP quantitative values. Graph created using Python (Python Software Foundation, Delaware, US).

Figure 7 summarizes the qualitative assessment of myocardial motion, confirming that the AFP metric constitutes a reliable indicator of data alignment quality.

## Discussion

T1 mapping techniques have consistently proven to be a reliable and useful diagnostic tool in cardiac imaging. T1 mapping is already established as a powerful technique for prognostic prediction in several cardiac conditions, such as in dilated cardiomyopathy^17^. But more importantly, T1 mapping derived parameters may be crucial for the recognition of cardiac involvement in several systemic and infiltrative disorders at early stages, when treatment options are expected to confer a major impact in clinical outcomes. For instance, T1 mapping can depict amyloid deposition in cases without any late gadolinium enhancement and has the potential to overcome some of the limitations for using gadolinium-based contrasts in such patients^18^. The technique is also gaining widespread use as a surrogate for the evaluation of cardiac response to treatment not only in amyloidosis, but also in other diseases, such us hemochromatosis or valvular heart diseases. The potential of this technique to track small but relevant changes in myocardial composition will have an enormous impact in the design of clinical trials in the next future. However, T1 mapping remains sensitive to the patient’s cardiac and respiratory motion, leaked into the acquisition due to triggering inaccuracies, arrhythmic heartbeats and breath-hold failures. The resulting alterations in estimated T1 values are not insignificant and need to be corrected to achieve results as accurate as possible.

Motion compensation techniques, usually in the form of post-reconstruction image registration, are often applied to improve the accuracy of the alignment^19^. El-Rewaidy et al. used a non-rigid active model-based registration framework^20^. Xue et al. used image registration with synthetic image estimation^21^. Perhaps the most similar approach to the one validated in this study is the one proposed by Tilborghs et al., which incorporated a T1 model in the registration pipeline^22^. The main differences between the proposed method and Tilborghs’ are driven by the need to increase computational efficiency and avoid reconstruction lag (i.e. that a reconstruction task is still ongoing when the acquisition of the next sequence completes). For this reason, the reference frame optimization is not performed in our case, multi-fitting is replaced with a single-fit method for phase determination and only two model-based iterations are applied. Also, manual definition of the heart ROI is not required and heart mask segmentation is skipped.

We have evaluated a new, dedicated MoCo algorithm for MOLLI T1 mapping series on a large set of clinical patients. Multiple instances of misalignment leading to partially inaccurate T1 maps have been identified in the CMR database collected for the present study. However, quantifying the improvement of T1 mapping with motion correction is not trivial, due to the lack of a reliable reference. Therefore, a quantitative data alignment criterion (AFP) has been defined, based on the coefficient of determination of the voxel-wise T1 fit in the cardiac region.

The resulting quantitative evaluation of motion correction results has shown that accounting for the relaxation of tissue in the registration similarity criterion significantly decreases the incidence of misregistration and subsequent T1 inaccuracies. As the results of the study show, the worst-case scenario where motion correction not only fails but causes a degradation of T1 map accuracy is greatly reduced with the proposed method, going from over 10% of cases to less than 1%.

Figure 5 provides a visual representation of the relative performance of the standard and proposed methods, in terms of the mapping accuracy improvement (or degradation) that they achieve compared to uncorrected data. While standard motion correction as implemented in the scanner shows improved mapping performance in a majority of cases, there is a sizeable proportion showing no gain or, in some cases, misregistration leading to inaccurate T1 values. In contrast, the proposed method improves mapping performance in all but a few cases, with an extensive majority achieving better results than the standard method, sustaining the affirmation that new method is superior in alignment accuracy and, above all, robustness. The small number of cases where the new motion correction method underperforms with respect to uncorrected data are typically cases without significant misalignment and cases moderate perceptual degradation (< 10%).

To confirm the validity of the quantitative evaluation criterion and derived results, a subset of the database has been reviewed using a qualitative data alignment score. This resulted in three goups of cases, corresponding to the three motion categories described in Table 1. The AFP means of these groups are significantly differentiated and correlated with the qualitative scale, validating the quantitative analysis. They are separated by approximately 5%, which gives an approximation of the perceptual impact of changes in the quantitative values. Two outliers appear in each of the three groups. These were individually reviewed and can be explained by non-motion artifacts and suboptimal slice orientations.

The results of the qualitative review confirm that the metric used for the quantitative analysis constitutes a valid surrogate of anatomical alignment and, by extension, of T1 mapping accuracy.

One limitation of the proposed method is appreciated in the reduced, but still present, number of cases where motion correction degrades data alignment in comparison to uncorrected data. A desirable feature would be the ability to either predict this outcome or detect it before the motion correction results are applied to generate the maps. Future work will focus on automating the quantiative data alignment criterion (currently relying on manual segmentation of the cardiac region) to identify potential motion correction failure cases.

## Conclusions

We have demonstrated a reconstruction pipeline with built-in motion correction, optimized for MOLLI T1 mapping sequences. The performance of the proposed method was compared to the standard motion correction implemented in the scanner, as well as to uncorrected reconstruction. A large database of clinical CMR cases has been used to perform quantitative and qualitative analysis of the reconstructions, confirming the increased accuracy and, more importantly, robustness of the T1 mapping results obtained with the new method.

## Supplementary Information

Supplementary Legends.

Supplementary Figure S1.

## Data Availability

The datasets during and/or analysed during the current study available from the corresponding author on reasonable request.

## Abbreviations

2D: Two-dimensional
AFP: Accurate fitting prevalence
ASSET: Array coil spatial sensitivity encoding
B0: Static magnetic field
bSSFP: Balanced steady-state free precession
CMR: Cardiovascular magnetic resonance
FOV: Field of view
GE: General Electric
MRI: Magnetic resonance imaging
MOLLI: Modified look-locker inversion recovery
R^2^: Coefficient of determination
T1: Spin–lattice relaxation time
TI: Inversion time
