# Supplementary figures and images for: Improving the robustness of MOLLI T1 maps with a dedicated motion correction algorithm
Source: Sci Rep. 2021 Sep 17;11:18546. doi: 10.1038/s41598-021-97841-z (PMC8448777; doi:10.1038/s41598-021-97841-z)

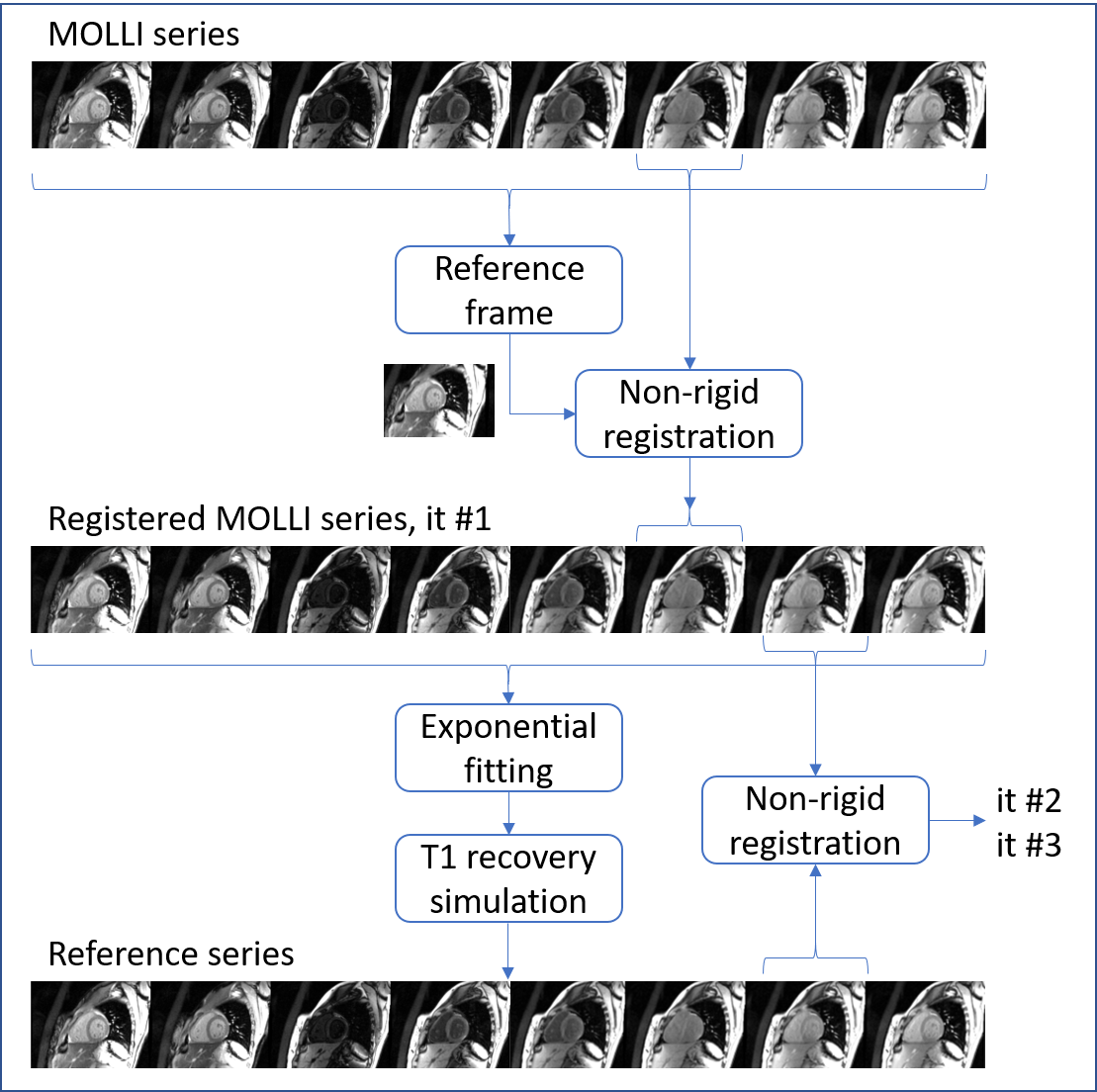

Supplement: Supplementary file 2 — Supplementary Figure S1. [file 41598_2021_97841_MOESM2_ESM.png]
